# Enhanced catalysis through structurally modified hybrid 2-D boron nitride nanosheets comprising of complexed 2-hydroxy-4-methoxybenzophenone motif

**DOI:** 10.1038/s41598-021-03992-4

**Published:** 2021-12-24

**Authors:** Pooja Rana, Ranjana Dixit, Shivani Sharma, Sriparna Dutta, Sneha Yadav, Aditi Sharma, Bhawna Kaushik, Pooja Rana, Alok Adholeya, Rakesh K. Sharma

**Affiliations:** 1 Green Chemistry Network Centre, Department of Chemistry, University of Delhi, New Delhi, 110007 India; 2 TERI-Deakin Nanobiotechnology Centre, TERI Gram, The Energy and Resources Institute, Gurugram, 122102 India

**Keywords:** Chemistry, Materials science, Nanoscience and technology

## Abstract

Tuning the structural architecture of the pristine two dimensional hexagonal boron nitride (*h*-BN) nanosheets through rational surface engineering have proven advantageous in the fabrication of competent catalytic materials. Inspired by the performance of *h*-BN based nanomaterials in expediting key organic transformations, we channelized our research efforts towards engineering the inherent surface properties of the exclusively stacked *h*-BN nanosheets through the incorporation of a novel competent copper complex of a bidentate chelating ligand 2-hydroxy-4-methoxybenzophenone (BP). Delightfully, this hybrid nanomaterial worked exceptionally well in boosting the [3 + 2] cycloaddition reaction of azide and nitriles, providing a facile access to a diverse variety of highly bioactive tetrazole motifs. A deep insight into the morphology of the covalently crafted *h*-BN signified the structural integrity of the exfoliated *h*-BN@OH nanosheets that exhibited lamellar like structures possessing smooth edges and flat surface. This interesting morphology could also be envisioned to augment the catalysis by allowing the desired surface area for the reactants and thus tailoring their activity. The work paves the way towards rational design of *h*-BN based nanomaterials and adjusting their catalytic potential by the use of suitable complexes for promoting sustainable catalysis, especially in view of the fact that till date only a very few *h*-BN nanosheets based catalysts have been devised.

## Introduction

**Figure 1:**
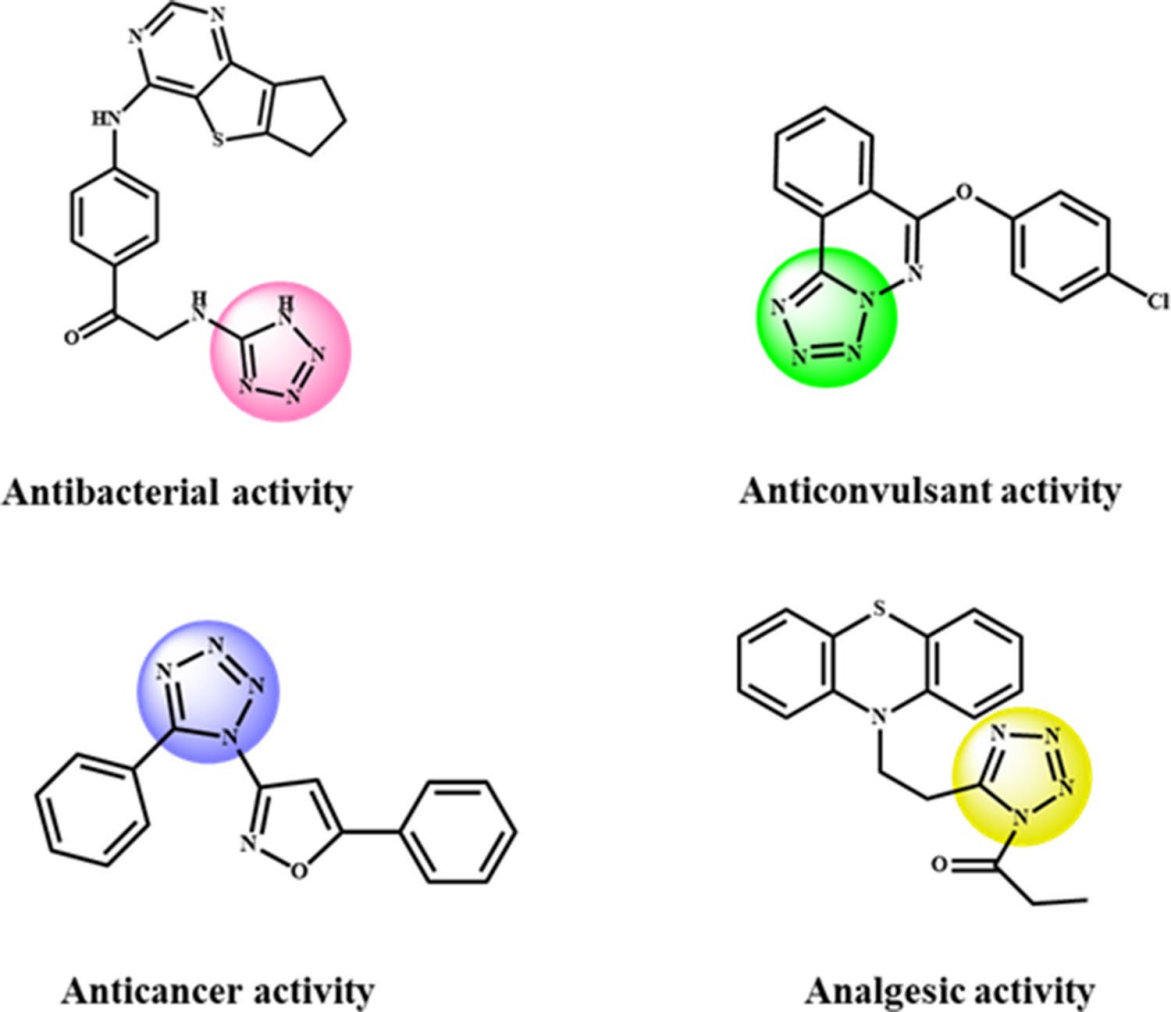
Biologically active molecules bearing tetrazole framework.

Two-dimensional hexagonal boron nitride nanosheets based architectures with long-range ordered atomic arrangements have recently stimulated the exponential growth in the arena of materials chemistry. Indeed, it is the exclusive stacked structure of BN nanosheets due to electronegativity difference between B and N atom which imparts several fascinating features such as excellent mechanical strength, outstanding thermal and chemical stability, low dielectric constant, oxidative resistance, nanometre size, large surface area to volume ratio and high complex loading^1–3^. Considering such intrinsic characteristics, research on structurally flexible *h*-BN based nanomaterials has been flourishing across the globe in myriad of diverse fields including sensing, electronics, sensors, hydrogen storage, gas separation, etc^4–8^. Very recently, these exotic materials have significantly garnered the attention of scientific community as a promising candidate to design new generation catalytic materials for cascade reactions due to their unique atomic structure. Notably, atomically thin *h*-BN nanosheets have received tremendous recognition as a solid matrix amongst various nanostructured materials to develop surface engineered catalysts as they are capable of dissipating considerable amount of heat in exothermic organic reactions^9,10^. Besides, they not only prevent catalyst deactivation by driving off the moisture owing to hydrophobic surface but also prevent the issue of silicates or aluminates formation often encountered in other oxide supports^11,12^. To date, catalytic efficacy of *h*-BN supported nanomaterials has been investigated in some organic reactions including oxidation of methane, benzene and alcohols, reduction of NOx, selective hydrogenation of unsaturated aldehyde and semi-hydrogenation of alkynes^13,14^. However, as evident from existing literature reports the potential of such 2D nanocomposites has not been explored in catalyzing cycloaddition of azide and nitriles. This industrially significant transformation furnishes 5-substituted 1*H*-tetrazoles which have gained tremendous impetus as active pharmaceutical ingredients since their pioneering discovery in year 1885 by Bladin due to their biological properties including antibiotic, anti-viral, anti-fungal, anti-cancer, anti-diabetic and anti-hypertensive agents (Fig. 1)^15–25^. Besides, these synthons also possess immense potential as promising candidates in coordination chemistry, photographic industry, agricultural field, organocatalysis and information recovery systems^26–31^. Such diverse applications of tetrazole derivatives and their inability to exist in nature have inspired various research groups worldwide to explore newer synthetic routes for their access^32,33^. Hantzsch and Vagt in 1901 attempted the first successful [3 + 2] cycloaddition of azide and nitriles to synthesize 5-substituted 1*H*-tetrazoles^34^. Thereafter, a plethora of homogeneous catalysts like bronsted acids, lewis acids, AlCl_3_, CdCl_2_, Fe(OAc)_2_, copper (I) chloride, etc. as well as a few heterogeneous catalytic systems have been reported for their synthesis in literature^35–38^. However, commercial utilization of aforementioned protocols is hindered due to innate shortcomings such as use of expensive metal salts, toxic solvents, prolonged reaction time, unsatisfactory yield, difficulty in separation and recovery of the catalyst^39–44^. In this perspective, advanced heterogenized nanocomposites comprising of homogeneous metal complexes immobilized on diverse support matrices, bearing uniform active sites similar to their homogeneous counterparts is highly desirable to expedite the concerned reaction^45,46^. Taking into consideration the scientific impression of these materials and in continuation of our ongoing research in the field of catalysis^47–53^, herein we demonstrate the fabrication of a surface engineered 2D hexagonal boron nitride supported copper (*h*-BN@APTES@BP@Cu) nanocomposite and investigation of its catalytic efficacy in the synthesis of tetrazole derivatives.

In fact, this is the first ever research study on cycloaddition of azide and nitriles catalyzed by two-dimensional *h*-BN based nanomaterial. As anticipated, the results of present work unveil that *h*-BN@APTES@BP@Cu besides exhibiting excellent catalytic performance in the concerned reaction is also capable of furnishing array of tetrazole derivatives under mild reaction conditions. However, it is worth mentioning that the native *h*-BN nanosheets do not themselves possess any significant catalytic activity as experimentally proven (Table S1, Supporting Information) but work as an appealing support material in the immobilization of the targeted metal complex. Further, it is envisaged that *h*-BN@APTES@BP@Cu can be employed as a high performance catalyst in expediting other industrially significant organic transformations in near future.

### Motivation and significance

This work is a step taken towards accomplishing the key goals of sustainable development that portray a strong vision of hope for humanity. The manipulation of *h*-BN to generate a surface engineered nanomaterial that works as a green catalyst for expediting the industrially significant cycloaddition reaction holds promising potential to completely revolutionize the chemical sector. The tethering of APTES has been accomplished for the very first time on this heteroatom containing boron nitride nanosheets which has been further attached to a novel ligand. The synergistic integration of unique properties of BN, Cu metal center and bidentate ligand in a single platform along with the unique schistose like morphology of the nanocatalyst conferring prospects of increased surface area have been envisaged to accelerate the performance of the catalyst (in terms of yield, reaction time and conditions).

## Results and discussion

### Catalyst fabrication

**Figure 2:**
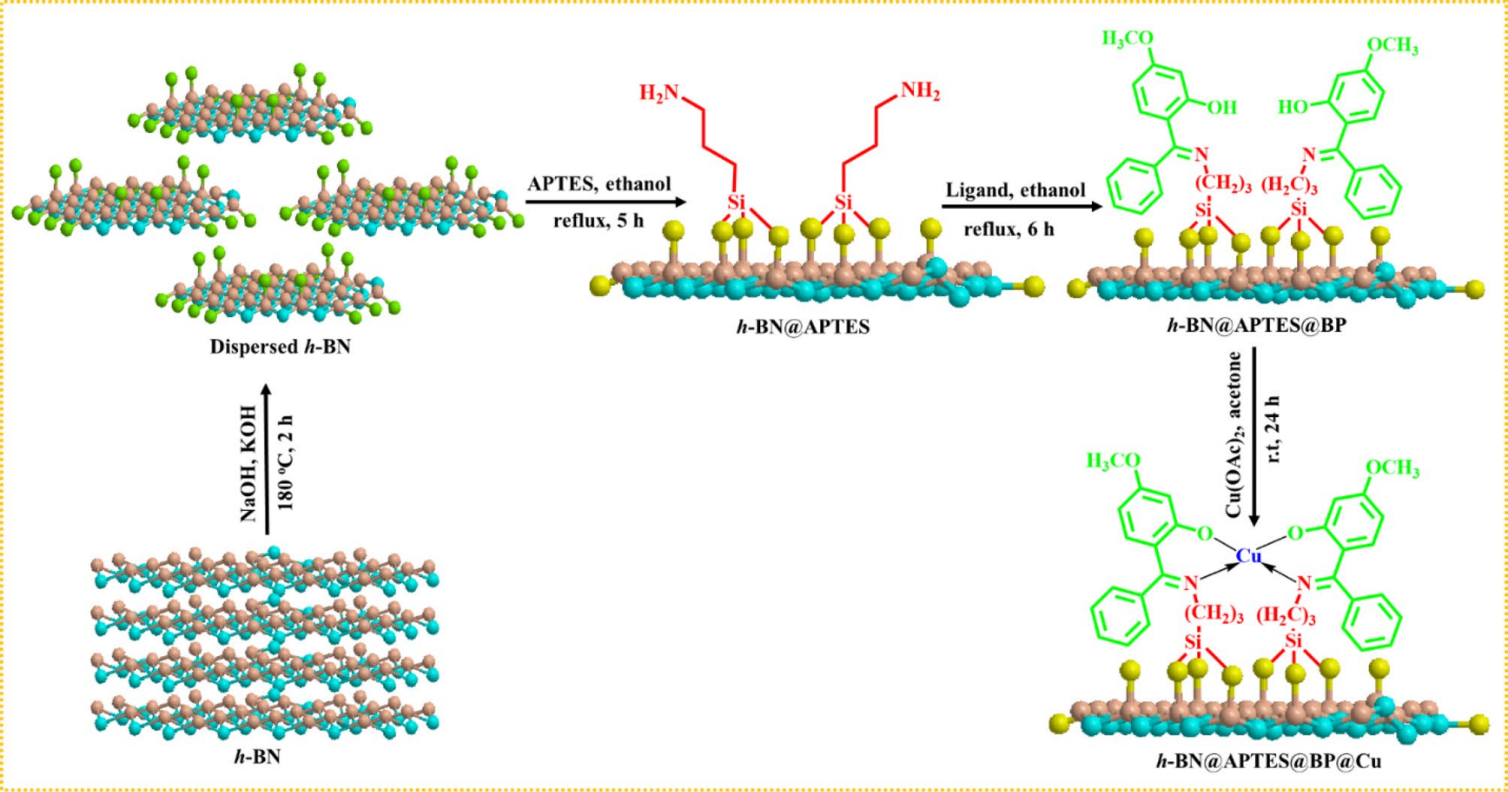
Fabrication of hexagonal boron nitride nanosheets based copper catalyst (*h*-BN@APTES@BP@Cu).

The catalyst has been fabricated in a stepwise manner through successive surface modifications of *h*-BN nanosheets, as displayed in Fig. 2. In the initial step, hydroxyl functionalized boron nitride (*h*-BN@OH) nanosheets were fabricated via ion-assisted liquid exfoliation approach by subjecting the homogeneous mixture of *h*-BN, sodium hydroxide (NaOH) and potassium hydroxide (KOH) to heating conditions in a stainless steel autoclave. A growth mechanism for the formation of *h*-BN nanosheets from bulk boron nitride micropowder is illustrated in Fig. S1, which comprises of molten alkali-assisted pretreatment and subsequent sonication. In molten alkali metal hydroxide treatment, ions such as Na^+^, K^+^ and OH^-^ are inserted into the interlayer space of the stacked sheets. In particular, these ions get adsorbed on the *h*-BN surface and then undergo diffusion into the space employed by adjacent BN lattices which results in the enlargement of interlayer spacing by weakening of adjacent layers held by van der Waals forces and curling of topmost BN sheet at the edges. As more number of ions get inserted, the curling up layer peels away from the parent counterpart by virtue of hydroxyl immobilized BN. Further, the resultant pretreated powder is subjected to liquid exfoliation under sonication which ensures high yield of *h*-BN nanosheets^54^.

Thereafter, the obtained *h*-BN@OH nanosheets were functionalized with amine moieties *i.e.* 3-aminopropyltriethoxysilane (APTES) under reflux condition in ethanol. This was done in order to generate the functional moieties on the surface of the nanosheets as amine groups are considered to be one of the most promising linkers that allow further scope for ready surface modification. Finally, *h*-BN@APTES@BP@Cu catalyst was synthesized by immobilizing 2-hydroxy-4-methoxybenzophenone onto the amine functionalized BN nanosheets (*h*-BN@APTES) via Schiff base condensation followed by metalation using copper acetate. The designed nanocatalyst was then characterized well using various physicochemical techniques such as scanning electron microscopy (SEM), transmission electron microscopy (TEM), fourier transform infrared (FT-IR), X-ray diffraction (XRD), energy-dispersive X-ray spectroscopy (EDS), energy-dispersive x-ray fluorescence (ED-XRF), Laser Raman spectroscopy and X-ray photoelectron spectroscopy (XPS).

### Catalyst characterizations

**Figure 3:**
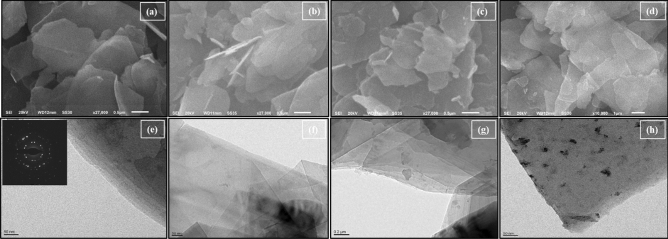
SEM micrographs of (**a**) *h*-BN@OH, (**b**) *h*-BN@APTES, (**c**) *h*-BN@APTES@BP, (**d**) *h*-BN@APTES@BP@Cu and TEM micrographs of (**e**) h-BN@OH, inset: corresponding SAED pattern, (**f**) *h*-BN@APTES, (**g**) *h*-BN@APTES@BP, (**h**) *h*-BN@APTES@BP@Cu.

SEM was employed to analyse the shape and surface morphology of the nanocomposites. The morphologies of *h*-BN@OH, *h*-BN@APTES, h-BN@APTES@BP and *h*-BN@APTES@BP@Cu using the technique and the resulting SEM micrographs have been provided in Fig. 3. The SEM micrograph of exfoliated *h*-BN@OH exhibits lamellar like structures comprising of smooth edges and flat surface. Further, on moving to *h*-BN@APTES, *h*-BN@APTES@BP and *h*-BN@APTES@BP@Cu, no morphological change is observed which suggests that the structural integrity remains unaltered even after functionalization. Besides SEM, TEM analysis of synthesized materials was also carried out which reveals layered like structure aligned in lateral dimension. TEM micrograph of *h*-BN@OH also shows 4–5 stacked sheets with average thickness lying between 3 and 7 nm. The typical six-fold symmetry of *h*-BN nanosheets was confirmed by selected area electron diffraction (SAED) pattern which further depicts its hexagonal crystal structure (Fig. 3e, inset). Moving to *h*-BN@APTES, *h*-BN@APTES@BP and *h*-BN@APTES@BP@Cu, no significant change in morphology is observed even after surface modification and successive metalation. However, the appearance of dark spots in the TEM micrograph of *h*-BN@APTES@BP@Cu can be attributed to the presence of Cu in the final catalyst.

FT-IR was performed to elucidate the stepwise synthesis of surface modified BN nanosheets as depicted in Fig. S2. The spectrum of *h*-BN@OH represents two intense peaks at 1372 and 820 cm^−1^ that are assigned to the B**−**N stretching and B** − **N** − **B bending vibration. Furthermore, an additional broad peak appearing at 3430 cm^−1^ confirms the presence of hydroxyl group on the surface of *h*-BN nanosheets as compared with the bulk *h*-BN. On moving to *h*-BN@APTES spectrum, emergence of bands at 1040 and 1120 cm^-1^ corresponds to the characteristic absorption of Si**−**O symmetric and asymmetric mode of vibrations which authenticates the existence of APTES moiety on the BN nanosheets surface through silylation process^55,56^. Additionally, absorption bands at 2936 and 1633 cm^-1^ are attributed to the CH_2_ and NH_2_ stretching vibrations of amino-propyl moiety. Furthermore, on moving to the FTIR spectra of *h*-BN@APTES@BP and *h*-BN@APTES@BP@Cu, no noticeable peak of C**=**N absorption (that usually is observed around 1632–1645 cm^−1^ due to the imine bond formation as a result of Schiff’s condensation between NH_2_ groups of *h*-BN@APTES and carbonyl groups of the ligand) can be seen which indicates that this peak is concealed under the broad band of B**−**N bonds^57,58^.

**Figure 4:**
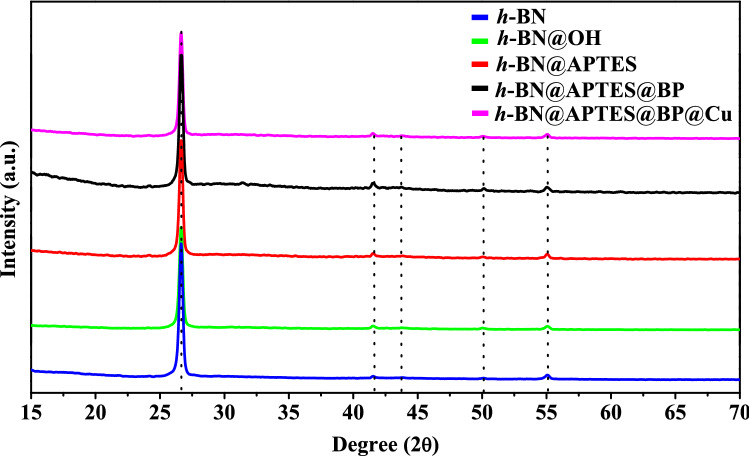
XRD spectra of *h*-BN, *h*-BN@OH, *h*-BN@APTES, *h*-BN@APTES@BP and *h*-BN@APTES@BP@Cu.

An insight into the crystalline behaviour of the designed nanocomposites was acquired through powder XRD analysis as shown in Fig. 4. XRD spectrum of *h*-BN@OH exhibits characteristic Bragg’s diffraction peak similar to the pristine *h*-BN powder at 2θ = 26.9°, 41.6°, 43.8°, 50.0° and 55.1° corresponding to the (002), (100), (101), (102) and (004) planes respectively (JCPDS card no. 34–0421)^59^. Further, similar diffraction peaks are obtained in the XRD spectra of *h*-BN@APTES, *h*-BN@APTES@BP and *h*-BN@APTES@BP@Cu which unveils no significant change in the crystallinity of nanosheets after being modified with the functionalizing agents. Moreover, no additional peaks corresponding to any other impurity is observed which indicates high purity of the sample.

**Figure 5:**
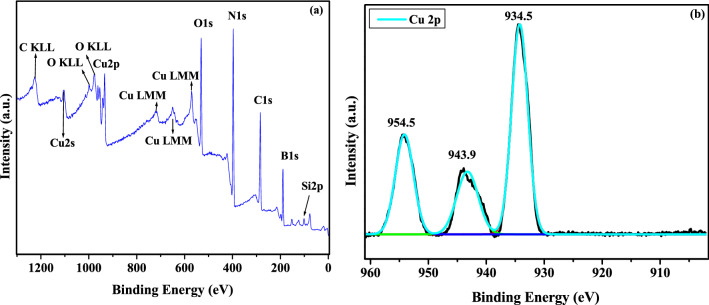
(**a**) Full range survey scan XPS spectra of *h*-BN@APTES@BP@Cu with Auger Cu LMM, O KLL and C KLL peaks and (**b**) core level spectrum of Cu 2p.

X-ray photoelectron spectroscopy was employed to investigate the surface electronic states of the developed catalyst. XPS survey spectrum of *h*-BN@APTES@BP@Cu and core level spectra of B 1 s, N 1 s, O 1 s, Si 2p and C 1 s elements are shown in Fig. 5a and Fig. S3 respectively^60,61^. In addition, a wide scan spectrum of *h*-BN@OH and corresponding core spectra of B 1 s, N 1 s and O 1 s are also provided in Fig. S4. As can be viewed from Fig. S3a, the appearance of two peaks located at 191.1 and 190.1 eV are attributed to the B**−**O and B**−**N bonds respectively^62^. Hence, it can be interpreted that −OH is attached to B atoms effectively rather than N atoms. The Si 2p spectrum (Fig. S3e) reveals strong peak at 102.1 eV which is accredited to the bond formation between silicon and oxygen (B**−**O**−**Si) and thus provides a strong evidence of silylation of the support material^63^. In N 1 s spectrum (Fig. S3b), an emerging peak at 397.8 eV is attributed to the binding energy of N-B bonds in *h*-BN nanosheets, whereas peaks observed at 398.6 and 399.1 eV are assigned to the N=C and N–H bonds, respectively^64,65^. Specifically, the peak corresponding to N=C authenticates the successful grafting of ligand onto the amine functionalized BN nanosheets via Schiff base condensation. In addition, the C 1 s spectrum of *h*-BN@APTES@BP@Cu (Fig. S3d) represents two bands amongst which band at 284.7 eV is attributed to the binding energy of C**=**C bonds and 286.7 eV is assigned to the C**−**O**−**C bonds^66^. Besides, the core level XPS spectrum of Cu 2p (Fig. 5b) displays two intense bands at 934.5 and 954.5 eV, which correspond to the binding energy of Cu(II) and an additional peak at 943.9 eV indicates the coordination linkage between copper and the ligand.

**Figure 6:**
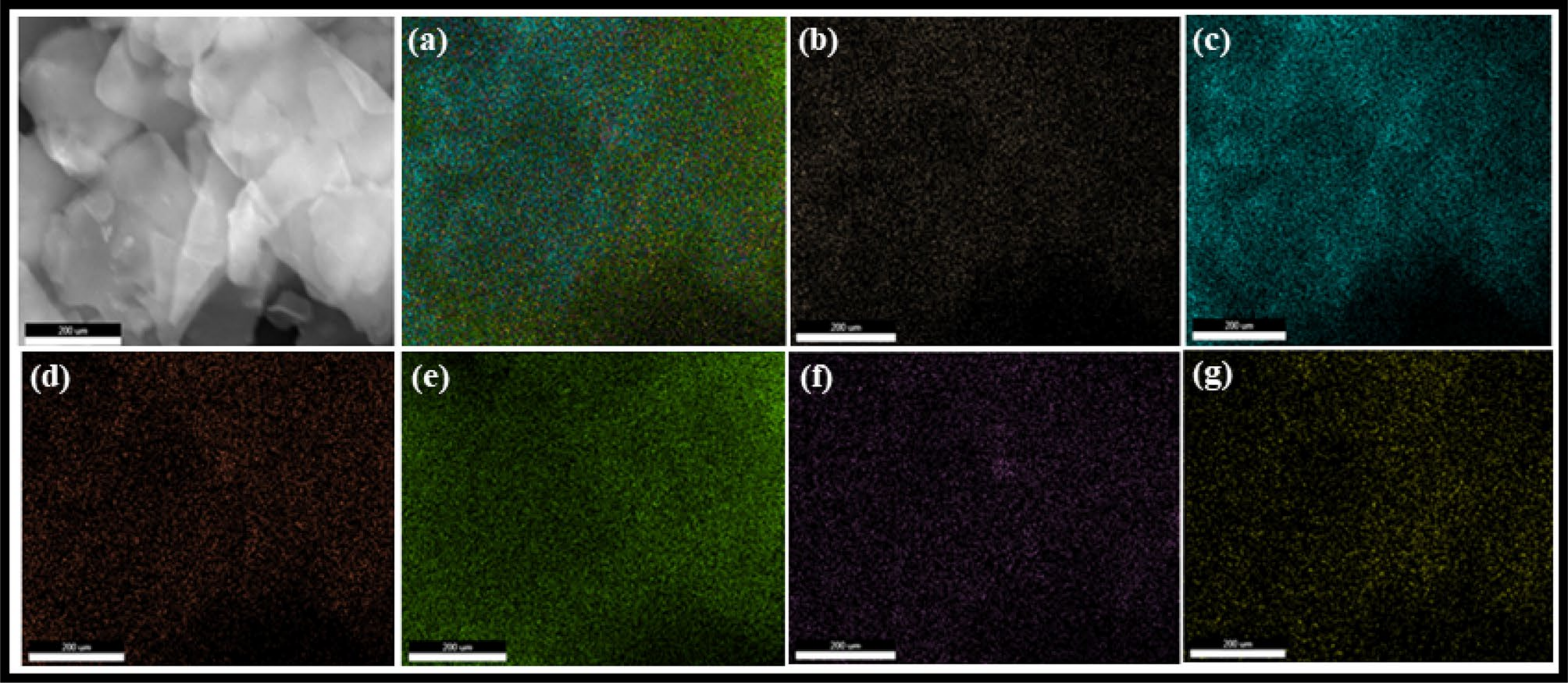
EDS elemental mapping images of (**a**) *h*-BN@APTES@BP@Cu showing uniform distribution of (**b**) B, (**c**) N, (**d**) O, (**e**) C, (**f**) Si and (**g**) Cu.

The elemental composition of the synthesized *h*-BN@APTES and *h*-BN@APTES@BP@Cu was confirmed by EDS (Fig. S5). The well-defined peaks of B, N, O, C and Si in Fig. S5a validates the anchoring of APTES onto the surface of BN nanosheets, while distinct peaks of B, C, N, O, Si and Cu in Fig. S5b corroborates the synthesis of *h*-BN@APTES@BP@Cu nanocatalyst. Moreover, well resolved peak of copper in the final nanocatalyst is also affirmed by ED-XRF spectroscopy (Fig. S6) which indicates successful introduction of metallic species on *h*-BN@APTES@BP. In addition, elemental mapping of *h*-BN@APTES@BP@Cu shows uniform distribution of B, N, O, C, Si and Cu elements in the final nanocatalyst (Fig. 6). Furthermore, the synthesized nanocatalyst was subjected to atomic absorption spectroscopy (AAS) to analyse the copper content and the corresponding loading was found to be 0.4878 mmol g^-1^.

**Figure 7:**
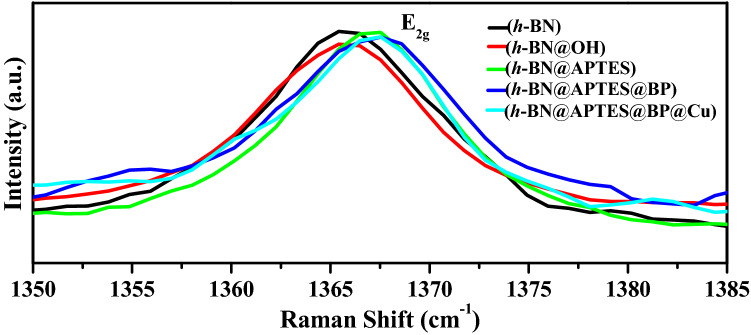
Raman spectra of *h*-BN, *h*-BN@OH, *h*-BN@APTES, *h*-BN@APTES@BP and *h*-BN@APTES@BP@Cu.

Raman spectrum of pristine BN shows characteristic G band at 1365 cm^-1^ corresponding to E_2g_ vibration mode (Fig. 7). Upon exfoliation, a blue shift to 1366 cm^-1^ is observed which indicates the formation of thinner flakes as a result of strong in-plane stresses and weak interlayer interaction^67–69^. Additionally, a decrease in peak intensity is also observed in case of *h*-BN nanosheets which provides strong evidence for the existence of highly exfoliated sheets compared to the bulk BN powder. On further modification of *h*-BN nanosheets surface, a blue shift in E_2g_ vibration mode is observed indicating smooth surface^70^.

### Catalytic evaluation

**Figure 8:**
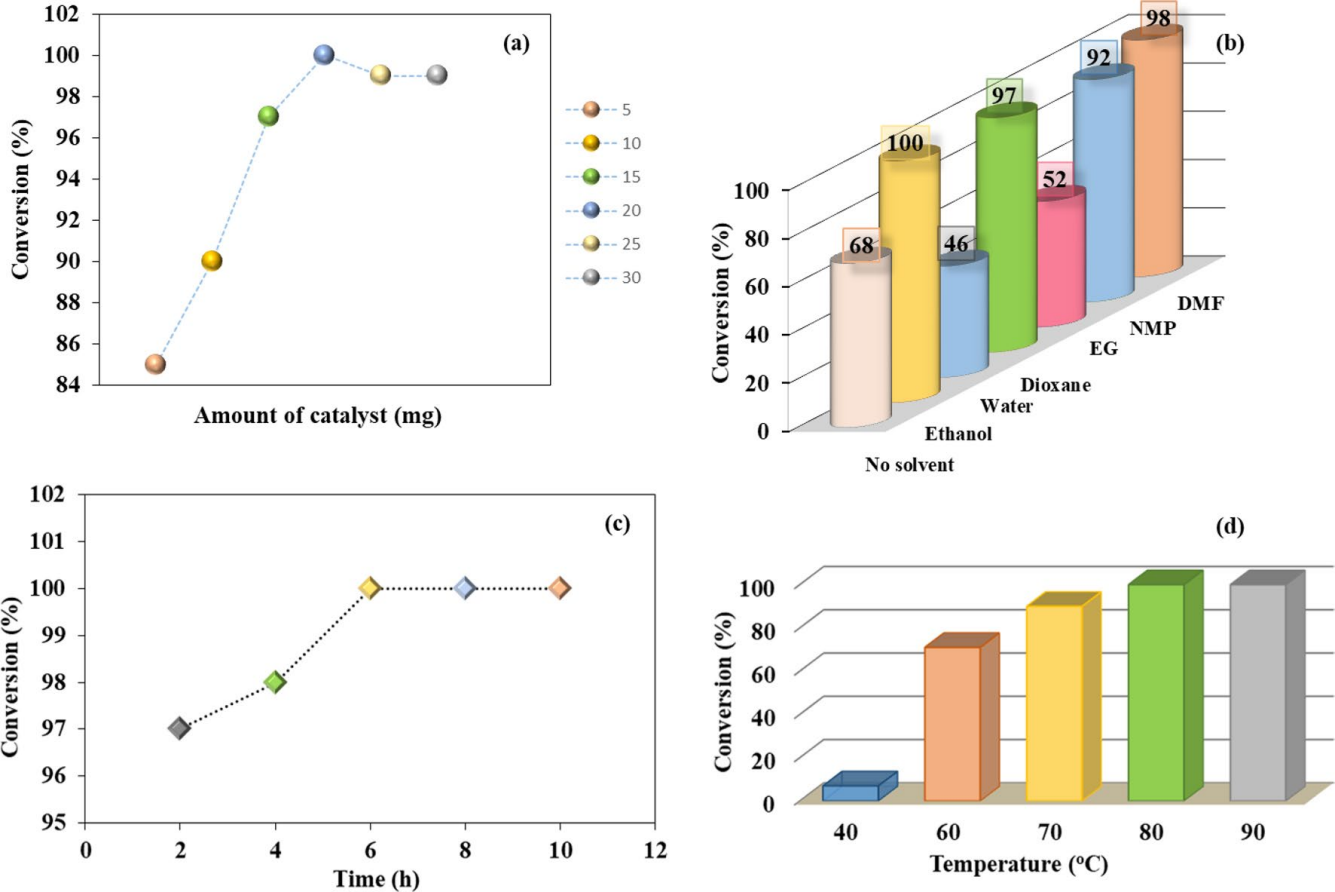
Effects of (**a**) amount of catalyst [reaction conditions: benzonitrile (1 mmol ), sodium azide (2 mmol), *h*-BN@APTES@BP@Cu (x mg), ethanol (1 mL), 80 °C, 6 h], (**b**) various solvents [reaction conditions: benzonitrile (1 mmol), sodium azide (2 mmol), *h*-BN@APTES@BP@Cu (20 mg), solvent (1 mL), 80 °C, 6 h], (**c**) time [reaction conditions: benzonitrile (1 mmol), sodium azide (2 mmol), *h*-BN@APTES@BP@Cu (20 mg), ethanol (1 mL), 80 °C] and (**d**) temperature variance on the cycloaddition of benzonitrile and sodium azide [reaction conditions: benzonitrile (1 mmol), sodium azide (2 mmol), *h*-BN@APTES@BP@Cu (20 mg), ethanol (1 mL), 6 h].

The catalytic potential of the newly fabricated *h*-BN@APTES@BP@Cu catalyst was examined in [3 + 2] cycloaddition of azide and corresponding nitriles leading to the synthesis of 5-substituted 1*H*-tetrazoles. To commence the investigation, benzonitrile and sodium azide were selected as test substrates. Moreover, various reaction parameters such as amount of catalyst, type of solvents, effect of time and temperature were determined to achieve an optimum reaction profile for the cycloaddition reaction with the aid of *h*-BN@APTES@BP@Cu (Fig. 8). A control experiment was carried out in the absence of catalyst using 1:2 ratio of test substrates (*i.e.* 1 mmol benzonitrile and 2 mmol sodium azide) which afforded trace amount of the desired product (Table S1). In addition, various homogeneous and heterogeneous catalysts were also deployed to afford the targeted product. Amongst all the tested catalytic materials, heterogeneous *h*-BN@APTES@BP@Cu presented highest conversion percentage and therefore endorsed its remarkable efficacy in the desired transformation.

The influence of variation in catalyst amount was also examined in the one-pot synthesis of tetrazoles. In this respect, six different sets of experiments were carried out by increasing the amount of catalyst from 5 to 30 mg (Fig. 8a). The results disclosed that on increasing the amount of catalyst from 5 to 20 mg, an increase in conversion percentage was observed due to increase in catalytic active sites. Further increase in catalyst loading led to a decrease in conversion percentage which could be attributed to the steric hindrance caused by low dispersity of excess catalyst. Therefore, optimum amount of *h*-BN@APTES@BP@Cu was found to be 20 mg which resulted in maximum conversion percentage.

Choice of solvent also plays a pivotal role in enhancing the catalytic efficacy of the reaction. In this context, model reaction was subjected to a series of solvents which included water, ethanol, ethylene glycol (EG), *N, N*-dimethylformamide (DMF), dioxane and *N*-methyl-2-pyrrolidone (NMP). The results revealed that the reaction proceeded with good conversion percentage using ethanol, dioxane, NMP and DMF solvents (Fig. 8b). Moreover, the reaction was also performed under solvent free conditions which showed low conversion percentage. Evidently, superior result was achieved when the reaction was conducted in ethanol. Hence, further optimizations were executed successfully using ethanol as a green solvent.

To determine the effect of time on the rate of reaction, model reaction was monitored at different time intervals ranging from 2 to 10 h. As shown in Fig. 8c, the conversion percentage is displayed as a function of time which demonstrated that maximum conversion percentage was observed when the reaction was allowed to run for 6 h. However, when the reaction proceeded further, no appreciable change was observed. Therefore, 6 h was considered as optimized time period for the cycloaddition of benzonitrile and sodium azide moieties.

In order to study the effect of temperature variance, test reaction was carried out at diverse range of temperature (40–90 °C) as presented in Fig. 8d. At 40 °C, conversion of the reactant to the desired product was found to be negligible. When temperature was increased to 60 °C, 71% of the product formation was achieved. Thereafter, an increase of 10 °C resulted in 90% conversion. The results revealed 100% conversion when temperature was raised to 80 °C. However, further rise in temperature resulted in no significant change in conversion percentage. Hence, the optimum temperature for [3 + 2] cycloaddition product was found to be 80 °C.

**Scheme 1:**
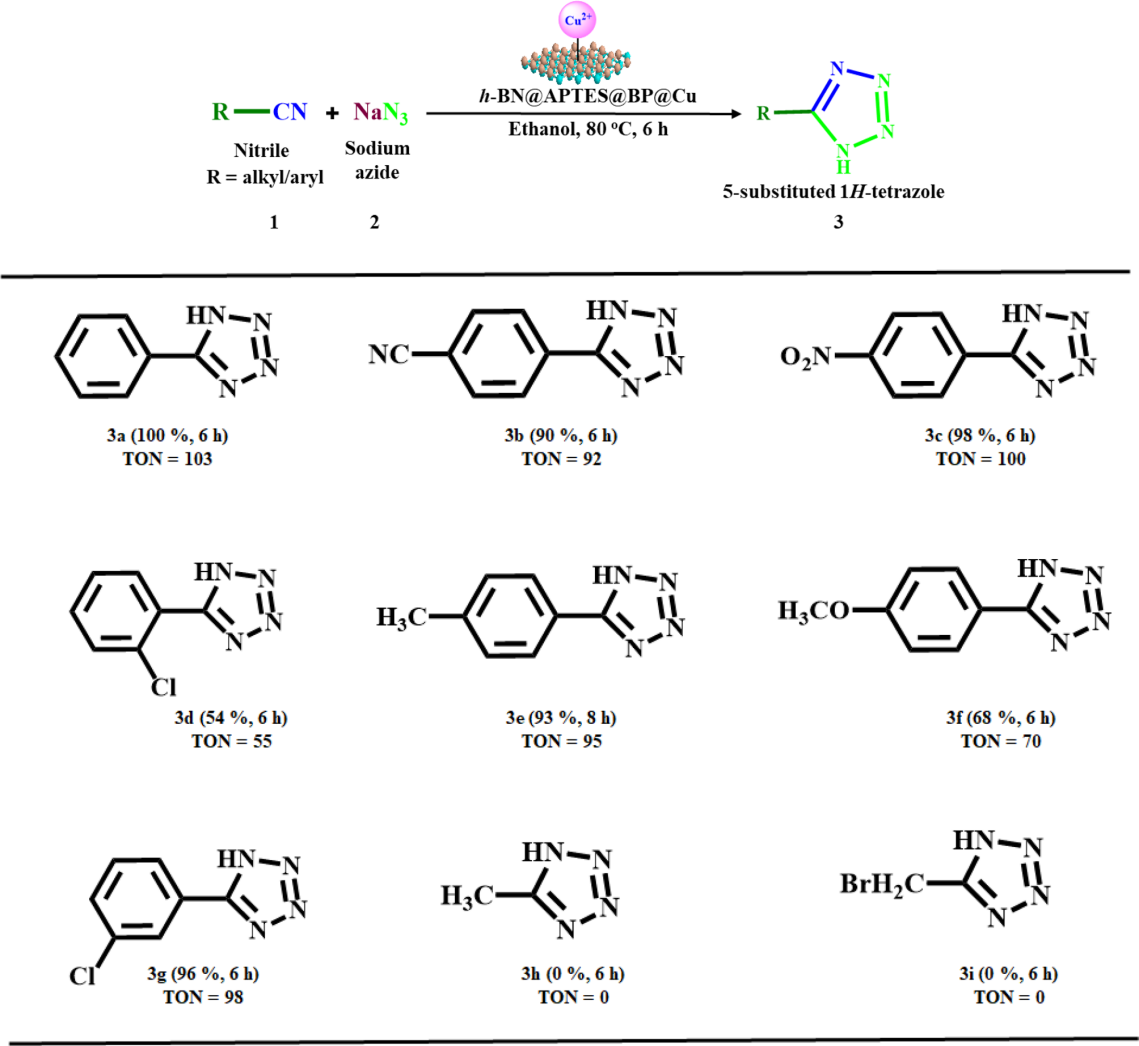
Synthesis of 5-substituted tetrazoles via [3 + 2] cycloaddition of nitriles and sodium azide^a^. ^a^Reaction conditions: Nitrile (1 mmol), sodium azide (2 mmol), *h*-BN@APTES@BP@Cu (20 mg) in ethanol (1 mL), 80 °C. Conversion percentages were determined via GC − MS. TON is the number of moles of the product per mole of the catalyst.

To explore the scope and applicability of this methodology, a series of benzonitriles were subjected to [3 + 2] cycloaddition reaction under the established ambient conditions. The concise results are summarized in Scheme 1. It was found that benzonitriles bearing both electron donating groups (entry 3e and 3f.) and electron withdrawing groups (entry 3b, 3c and 3 g) furnished corresponding tetrazoles in moderate to excellent conversion percentage. In particular, superior results were obtained in case of nitriles possessing electron withdrawing groups. This could be attributed to –I effect of the substituents that makes the benzonitrile more electrophilic thereby activating the benzonitrile towards nucleophilic attack via azide ion. However, nitriles that comprise of electron donating substituents proceeded with relatively longer time period (entry 3e). Besides, the steric hindrance caused by chlorine group at *ortho* position resulted in lower conversion percentage (entry 3d). Moreover, aliphatic nitriles were also subjected to the optimized reaction conditions. Unfortunately, the desired products were not obtained (entry 3 h and 3i).

**Figure 9:**
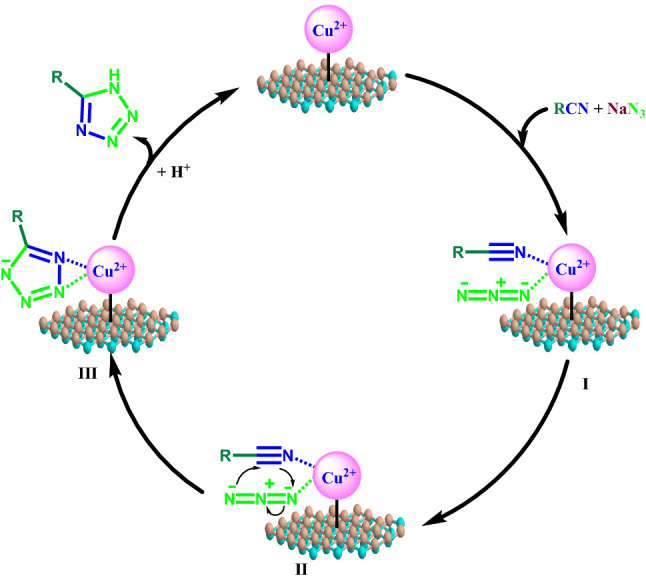
Plausible mechanism for the cycloaddition reaction catalyzed by *h*-BN@APTES@BP@Cu nanocatalyst.

On the basis of literature precedents, a plausible reaction pathway has been proposed to synthesize 5-substituted 1*H*-tetrazoles using *h*-BN@APTES@BP@Cu catalyst as outlined in Fig. 9.^39^ Initially, coordination of nitrogen atoms of both the nitrile and azide moieties with Cu (II) generates complex I that accelerates the [3 + 2] cyclization step as shown in complex II wherein subsequent nucleophilic attack of azide ion onto the nitrile group leads to the formation of complex III. Thereafter, acidic work-up protonates the complex III which results in the formation of desired tetrazole product with the release of catalyst. The structural integrity of the recovered catalyst remained unaltered even after being reused for several runs.

A leaching experiment was also performed using hot filtration method in order to certify heterogeneity of the catalyst. Thus, the test reaction was carried out under optimized reaction conditions using *h*-BN@APTES@BP@Cu catalyst. After half the reaction time, the catalyst was removed from the reaction mixture. The resulting supernatant was allowed to react further for appropriate period of time. GC–MS results displayed no significant increment in the conversion percentage of 5-substituted 1*H*-tetrazole which debarred the possibility of leaching of active metal species from its solid support. Therefore, it could be interpreted that the copper complex remained intact onto the solid material which provided a strong evidence for the heterogeneous character of the catalyst.

**Figure 10:**
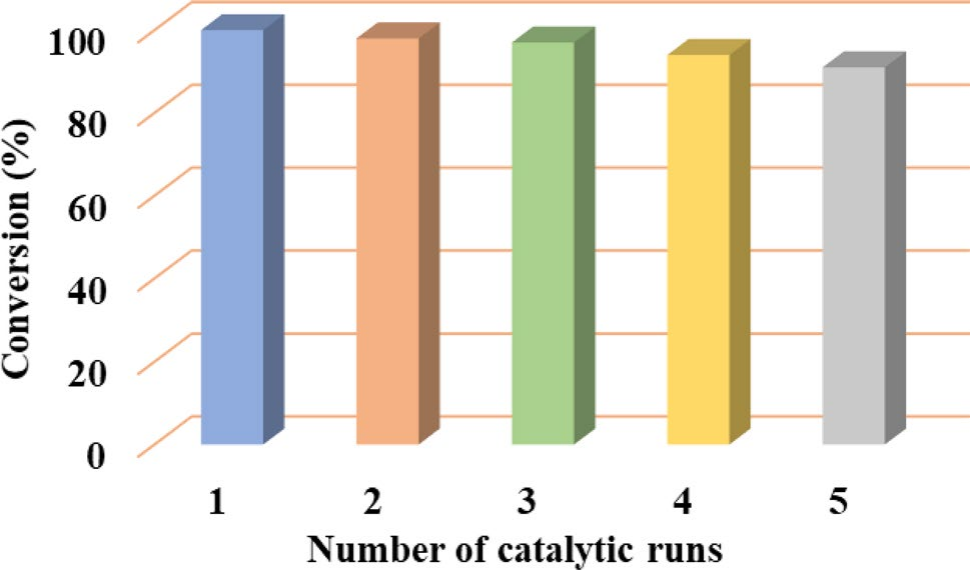
Recycling experiment for the cycloaddition of benzonitrile and sodium azide [reaction conditions: benzonitrile (1 mmol), sodium azide (2 mmol), *h*-BN@APTES@BP@Cu (20 mg) in ethanol (1 mL), 80 °C, 6 h].

The recyclability of *h*-BN@APTES@BP@Cu was examined under the optimized reaction conditions using benzonitrile and sodium azide as model substrates (Fig. 10). After completion of the reaction, the catalyst was retrieved by means of centrifugation, washed with ethyl acetate to remove residue of the reaction mixture and eventually dried well under vacuum. The recovered catalyst was then used for successive cycles by maintaining similar experimental conditions. The results authenticated that *h*-BN@APTES@BP@Cu could be reused efficaciously for five consecutive runs with no obvious deterioration in its catalytic activity. Further, on comparing SEM spectra of the recovered catalyst with the freshly prepared catalyst, no remarkable changes in shape and morphology was observed (Fig. S7). Additionally, XRD spectrum of the recovered catalyst showed identical Bragg’s diffraction peaks corresponding to the (002), (100), (101), (102), and (004) planes when compared with the freshly prepared catalyst (Fig. S8). These results provided a concrete evidence of the excellent durability of the synthesized BN supported copper catalyst.

To date, various homogeneous as well as heterogeneous catalysts have been utilized for the one-pot synthesis of tetrazoles. As evident from the Table S2, *h*-BN@APTES@BP@Cu nanocatalyst showed its superiority over previously reported homogeneous and heterogeneous catalysts in terms of product yield, reaction conditions and recyclability. The previously reported homogeneous catalyst underwent decomposition immediately after the completion of reaction thereby creating separation problems and thus could not be reused for further consecutive runs. In contrast, the present catalyst could be retrieved and reused for several runs without any remarkable loss in its catalytic activity. Moreover, the use of ethanol as a green solvent rendered this protocol economic and environmentally benign. Conclusively, as compared to the previously reported heterogeneous catalytic systems, *h*-BN@APTES@BP@Cu exhibited higher yield, mild reaction conditions and good recyclability.

## Conclusion

The present research discloses the design and fabrication of a novel exclusively stacked *h-*BN supported copper nanocatalyst (*h*-BN@APTES@BP@Cu) obtained via the covalent tethering of a bidentate 2-hydroxy-4-methoxybenzophenone ligand onto the amine functionalized support, accompanied by the metallation. The resulting nanomaterial unveiled exceptional catalytic performance in the cycloaddition of azide and nitrile to form biologically demanding and pharmaceutically important 5-substituted 1*H*-tetrazole scaffolds. The catalytic protocol adhered to the key goals of sustainable chemistry by fundamentally relying on the use of ethanol as the solvent that has been demarcated as “green." Some of the other salient features of this methodology included wider functional group tolerance, high turnover number, shorter reaction time and low temperature conditions, no use of additives, good recoverability and recyclability (up to 5 consecutive cycles). Additionally, the catalyst design approach principally relied on a covalent grafting approach that debarred any possibility of leaching as evidenced through the leaching test; supporting and signifying the durability of the engineered *h*-BN material, unlike many of the previously utilized catalysts for the tetrazole synthesis. We anticipate that this work will enthuse the scientific community, providing them the wisdom required for the design of efficient *h*-BN based catalytic materials through rational surface engineering in order to further increase their adaptability towards key industrial reactions.

## Experimental

### Materials and reagents

Boron nitride micropowder (Alfa Aesar), 3-aminopropyltriethoxysilane (APTES, Sigma-Aldrich), and 2-hydroxy-4-methoxybenzophenone (Spectrochem Pvt. Ltd.) were commercially procured. All other starting materials and reagents required in the study were purchased from Alfa Aesar and Spectrochem Pvt. Ltd.

### Instrumentation

The information about the crystallographic structure of the nanocomposites was estimated by powder X-ray diffraction (XRD) using a Bruker, D8 Advance (Karlsruhe, Bundesland, Germany) diffractometer equipped with Cu/Kα radiation at a scanning rate of 4° min^−1^ in the 2θ range of 15–70° (λ = 0.15405 nm, 40 kV, 40 mA). Fourier transform infrared (FT-IR) spectra were obtained using Bruker Alpha II in KBr mode. The spectra were operated in the transmission range of 4000 − 500 cm^−1^ under atmospheric conditions. The morphology and shape of nanosheets were determined by Jeol scanning electron microscope (SEM) spectroscopy, wherein preparative steps involved loading of small amount of sample on a carbon tap followed by coating with a thin layer of platinum using a sputter coater. The size of the developed nanocomposites was investigated through transmission electron microscopy (TEM), FEI TECHNAI G^2^ T20 at 200 kV. Energy-dispersive X-ray spectroscopic (EDS) analysis (equipped with the SEM instrument) was performed for the elemental mapping of the nanocomposites. Energy-dispersive X-ray fluorescence (ED-XRF) spectroscopy was also employed using a Fischerscope X-ray XAN-FAD BC. Laser Raman measurements were performed using RENISHAW, INVia. The amount of copper present in the catalyst was determined through a flame atomic absorption spectroscopy (model no. N3180021 PinAAcle 500) using acetylene flame. Temperature and pressure equipped instrument (Anton Paar Multiwave 3000) was used for the microwave-assisted digestion of the catalyst. The derived products were analysed and confirmed through the gas chromatography-mass spectroscopy (GC–MS) hyphenated technique that was conducted using an Agilent gas chromatograph (6850 GC) with a HP-5MS 5% phenyl methyl siloxane caplillary column (30.0 m × 250 μm × 0.25 μm) and a quadrupole mass filter equipped with 5975 mass selective detector (MSD) using helium as a carrier gas.

### Fabrication of boron nitride nanosheets (*h*-BN@OH)

Hydroxyl functionalized boron nitride nanosheets were synthesized using ion-assisted liquid exfoliation method^71^. In a typical synthesis, NaOH (2.84 g) and KOH (2.16 g) were finely ground followed by addition of *h*-BN micropowder (1.0 g) to obtain a homogeneous mixture and then transferred to a teflon-lined stainless steel autoclave. The mixture was heated at 180 °C for 2 h. Thereafter, the suspension collected from the autoclave was cooled down to room temperature and dispersed in deionized water (300 mL) under sonication for a time period of 30 min. The resultant nanosheets were then separated via centrifugation and washed with deionized water several times to remove excess hydroxides and other unreacted materials. After centrifugation, the product was dried well under vacuum overnight.

### Synthesis of amine functionalized BN nanosheets (*h*-BN@APTES)

Amine functionalized *h*-BN were synthesized using a previously reported method with slight modifications^58^. In particular, APTES (2 mmol) was added dropwise to a well-dispersed *h*-BN@OH (1 g) solution in ethanol (50 mL) and stirred under reflux condition for 5 h. The resulting *h*-BN@APTES solid was separated via centrifugation, washed thoroughly with ethanol to eliminate the unreacted silylating agent and finally dried under vacuum.

### Synthesis of boron nitride nanosheets supported copper catalyst (*h*-BN@APTES@BP@Cu)

The final catalyst *h*-BN@APTES@BP@Cu was prepared in two steps beginning from the synthesis of ligand grafted boron nitride nanosheets followed by its metalation. Firstly, 2-hydroxy-4-methoxybenzophenone (BP, 10 mmol) was added to 50 mL ethanolic *h*-BN@APTES (0.5 g) solution and the mixture was refluxed for 6 h. Thereafter, the solid product was separated by centrifugation and washed with ethanol^50^. In the next step, *h*-BN@APTES@BP was added to the copper acetate solution in acetone with uniform stirring at room temperature for a period of about 24 h. Further, the resulting nanocatalyst (*h*-BN@APTES@BP@Cu) was centrifuged, washed thoroughly with acetone and dried under vacuum at 60 °C^72^.

### General procedure for the synthesis of tetrazoles using boron nitride supported copper nanocatalyst (*h*-BN@APTES@BP@Cu)

The synthesis of desired tetrazole moiety involves the reaction between benzonitrile and sodium azide. For this, benzonitrile (1 mmol) and sodium azide (2 mmol) were mixed under stirring in a 25 mL round bottom flask containing 1 mL of ethanol followed by the addition of *h*-BN@APTES@BP@Cu (20 mg). The resulting mixture was refluxed for 6 h. After completion of reaction, the mixture was allowed to cool down to room temperature and the catalyst was retrieved by centrifugation. Consequently, HCl (5 N) was added to the resulting mixture and was extracted with ethyl acetate. Thereafter, combined organic layers were separated and dried over anhydrous sodium sulphate. Finally, the product obtained was confirmed by gas chromatography mass spectroscopy (GC–MS).

## Supplementary Information

Supplementary Information.
