# Supplementary material for: Enhanced catalysis through structurally modified hybrid 2-D boron nitride nanosheets comprising of complexed 2-hydroxy-4-methoxybenzophenone motif
Source: Sci Rep. 2021 Dec 24;11:24429. doi: 10.1038/s41598-021-03992-4 (PMC8709843; doi:10.1038/s41598-021-03992-4)
Supplement: Supplementary file 1 — Supplementary Information. [file 41598_2021_3992_MOESM1_ESM.docx]

**Enhanced catalysis through structurally modified hybrid 2-D boron nitride nanosheets comprising of complexed 2-hydroxy-4-methoxybenzophenone motif**

**Pooja Rana^1^, Ranjana Dixit^1^, Shivani Sharma^1^, Sriparna Dutta^1^, Sneha Yadav^1^, Aditi Sharma^1^, Bhawna Kaushik^1^, Pooja Rana^1^, Alok Adholeya^2^* and Rakesh K. Sharma^1^***

^1^ Green Chemistry Network Centre, Department of Chemistry, University of Delhi, NewDelhi-110007, India. Fax: +91-011-27666250; Tel: 011-276666250 Email: [rksharmagreenchem@hotmail.com](mailto:rksharmagreenchem@hotmail.com)

^2^TERI-Deakin Nanobiotechnology Centre, TERI Gram, The Energy and Resources Institute, Gurugram 122102, India

**Table of Contents**

| **S. No.** | **Content** | **Page No.** |
| --- | --- | --- |
| 1. | **Results and Discussion** | **S3** |
|  | Catalyst fabrication | **S3** |
|  | FT-IR analysis | **S4** |
|  | XPS analysis | **S5-S6** |
|  | EDS | **S7** |
|  | ED-XRF | **S8** |
| 2. | **Tables** | **S8** |
|  | **Table S1** Screening of various catalysts for the synthesis of tetrazoles | **S8-S9** |
|  | **Table S2** Comparison of the catalytic activity of *h*-BN@APTES@BP@Cu with previously reported protocols | **S9-S10** |
| 3. | **Recyclability tests** | **S10-S11** |
| 4. | **GC-MS spectra** | **S12-S25** |

**Results and Discussion**

**Catalyst fabrication**


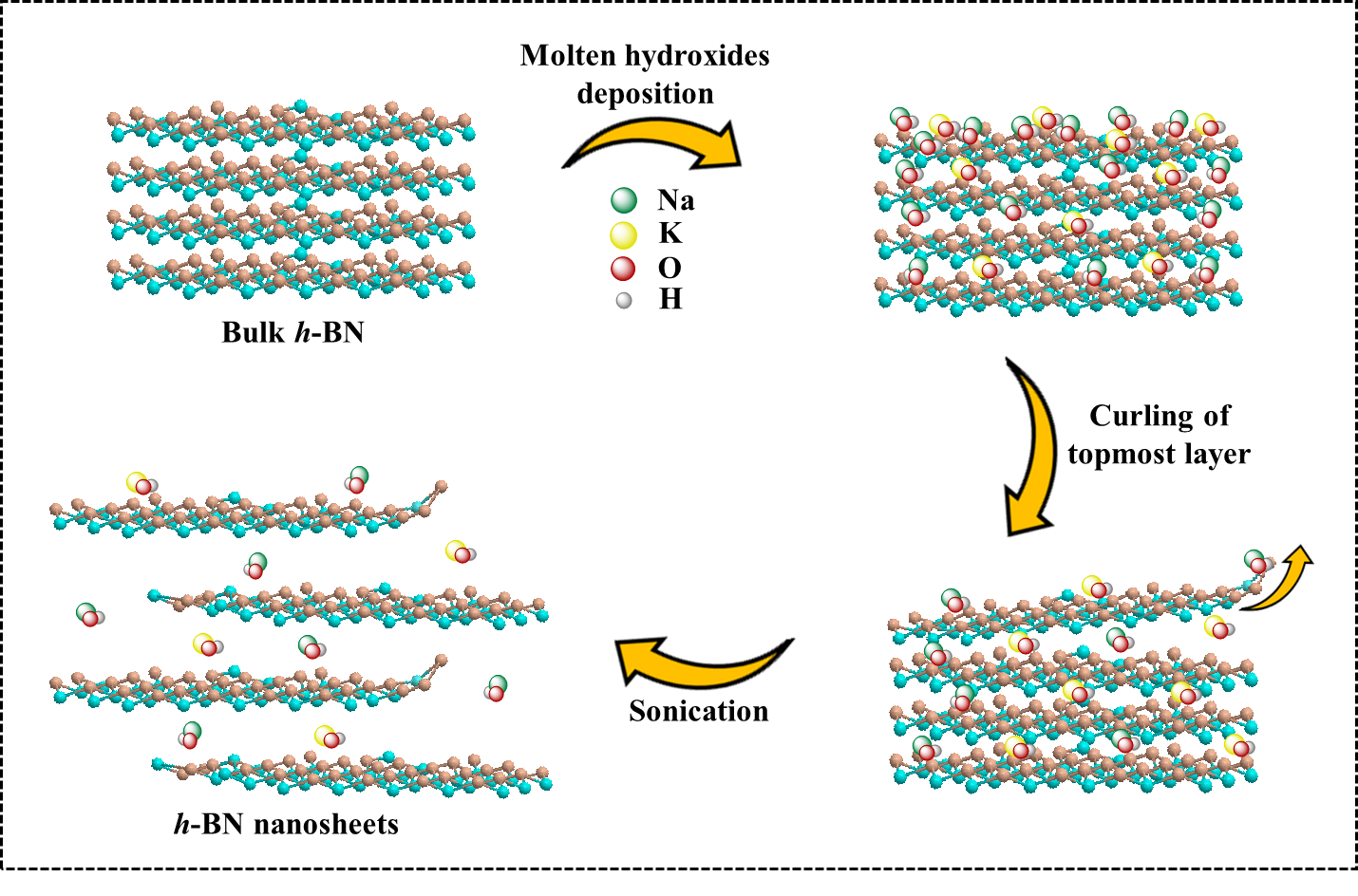


**Figure S1.** A mechanistic approach for the exfoliation of bulk *h*-BN.

**FT-IR analysis**

**Figure S2.** FT-IR spectra of (a) *h*-BN, (b) *h*-BN@OH, (c) *h*-BN@APTES, (d) *h*- BN@APTES@BP and (e) *h*-BN@APTES@BP@Cu.

**XPS analysis**

**
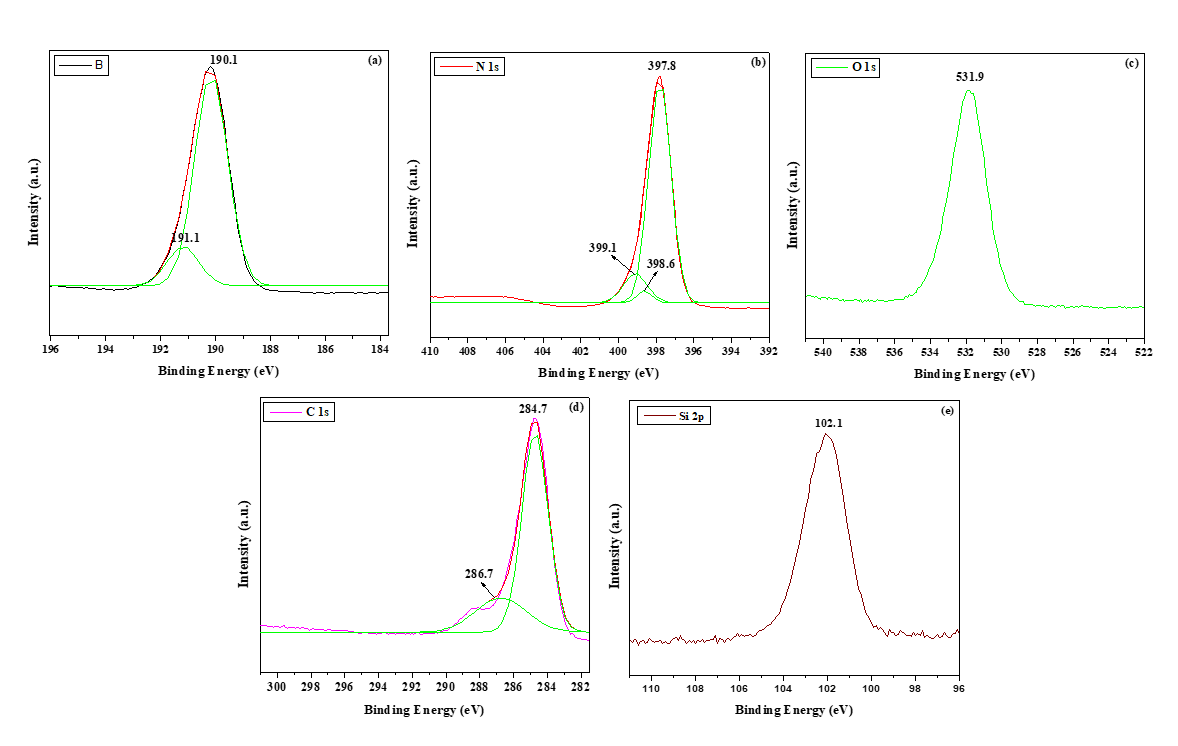
**

**Figure S3.** Core level XPS spectra of (a) B 1s, (b) N 1s, (c) O 1s, (d) C 1s and (e) Si 2p in *h*-BN@APTES@BP@Cu.


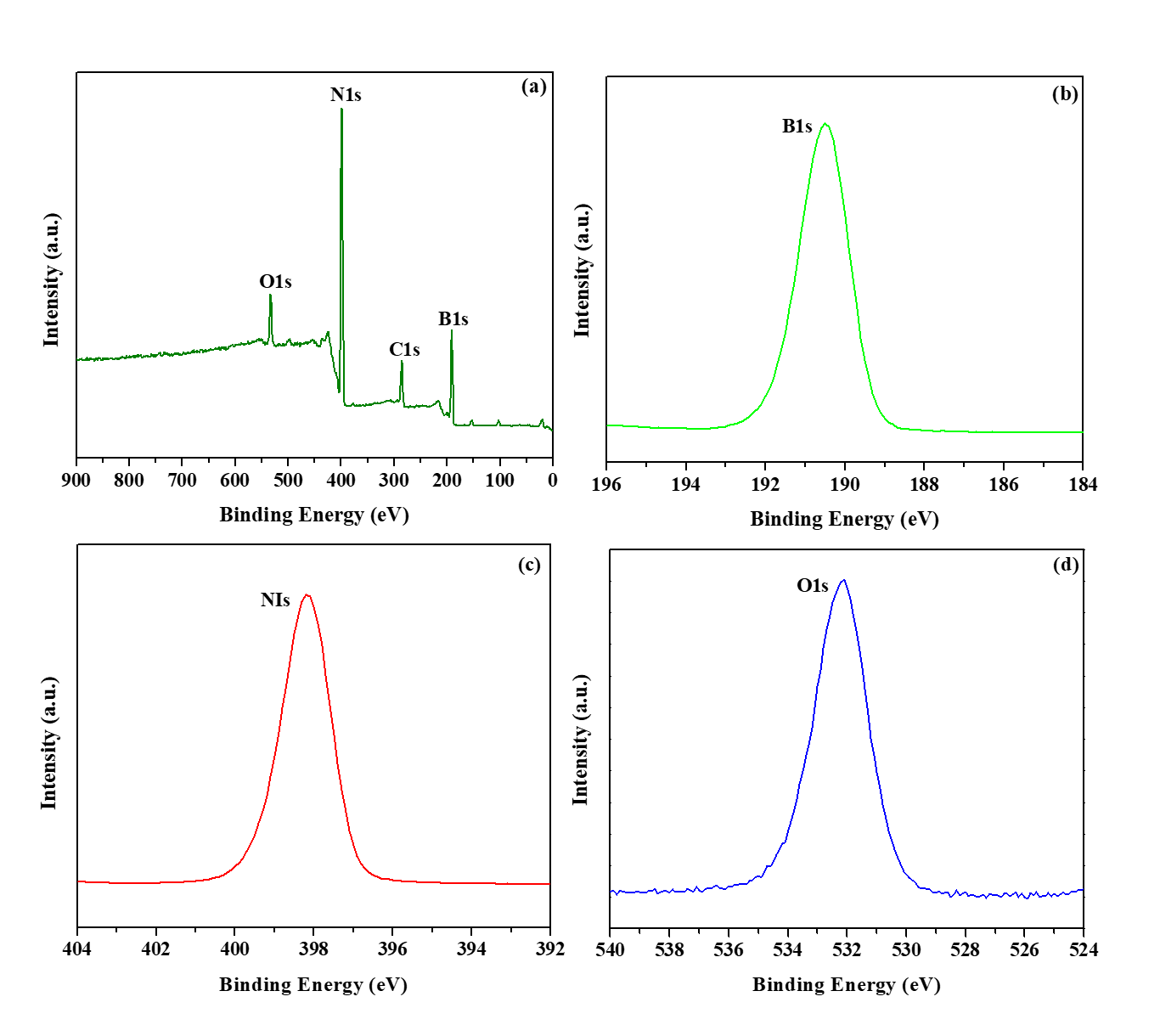


**Figure S4.** Wide scan XPS spectrum of (a) h-BN@OH, core-level XPS spectra of (b) B 1s, (c) N 1s and (d) O 1s.

**EDS**

**
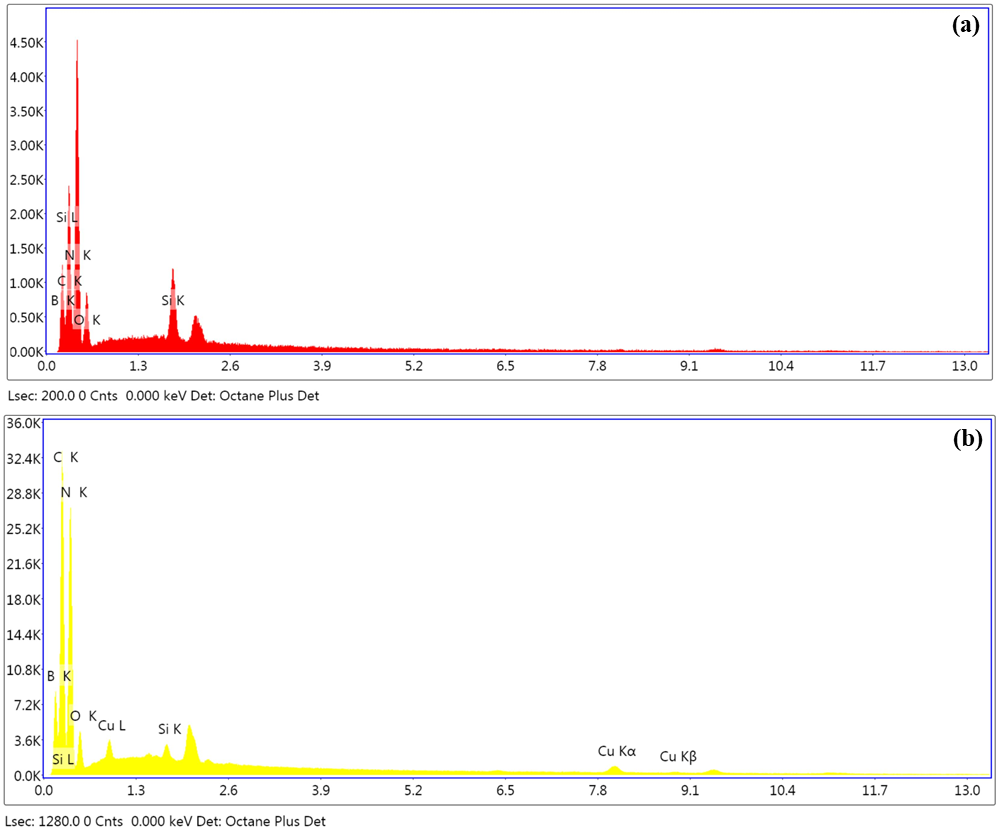
**

**Figure S5.** The EDS spectra of (a) *h*-BN@APTES and (b) *h*-BN@APTES@BP@Cu.

**ED-XRF**


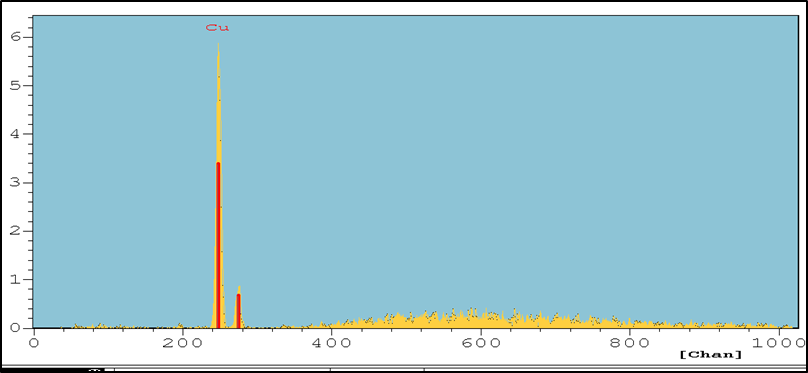


**Figure S6.** ED-XRF spectrum of *h*-BN@APTES@BP@Cu.

**Table S1.** Screening of various catalysts for the synthesis of 5-phenyl 1*H*-tetrazoles *via* [3+2] cycloaddition of benzonitrile and sodium azide^a^.

**
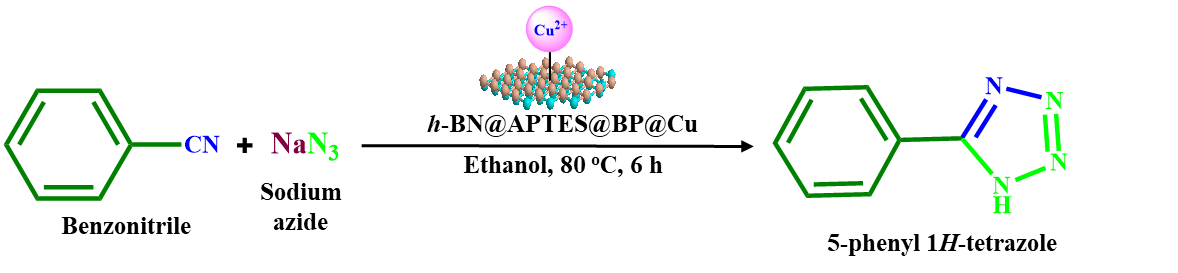
**

| **Entry** | **Catalyst** | **Conversion % ^b^** |
| --- | --- | --- |
| 1. | No catalyst | Trace amount |
| 2. | Cobalt chloride | 93 |
| 3. | Nickel chloride | 86 |
| 4. | *h*-BN@OH | Trace amount |
| 5. | *h*-BN@APTES | Trace amount |
| 6. | *h*-BN@APTES@BP | Trace amount |
| 7. | *h*-BN@APTES@BP@Cu | 100 |

**^a^Reaction conditions:** benzonitrile (1 mmol), sodium azide (2 mmol), catalyst (20 mg), ethanol (1 mL), 6 h, 80 ^o^C. ^b^Conversion percentage were determined *via* GC-MS.

**Table S2.** Comparison of the catalytic activity of *h*-BN@APTES@BP@Cu nanocatalyst with other previously reported catalysts for [3+2] cycloaddition of benzonitrile and sodium azide.

| **Entry** | **Aryl nitrile** | **Sodium azide** | **Catalytic conditions** | **Reusable cycles** | **Yield (%)** | **Ref.** |
| --- | --- | --- | --- | --- | --- | --- |
| 1. |  | NaN_3_ | CoY, DMF, 120 ^o^C, 14 h | 5 | 90 | 39 |
| 2. |  | NaN_3_ | 3D Porous ZnO, DMF, 125 °C, 14 h | 4 | 87 | 40 |
| 3. |  | NaN_3_ | ZnO/Co_3_O_4_, DMF, 120 ^o^C, 12 h | 5 | 90 | 41 |
| 4. |  | NaN_3_ | β-CD, NH_4_Cl, DMF, 120 °C, 0.75 h | 4 | 89 | 42 |
| 5. |  | NaN_3_ | Pt NPs@GO, DMF, 75 °C, 2 h | 6 | 96 | 43 |
| 6. |  | NaN_3_ | NiFe_2_O_4_ NPs, NH_4_OAc, DMF, 100 °C, 3 h | 5 | 94 | 44 |
| 7. |  | NaN_3_ | Cu_2_(BDC)_2_(DABCO), DMF, 120 °C, 1 h | 5 | 93 | 45 |
| 6. |  | NaN_3_ | *h*-BN@APTES@BP@Cu, ethanol,  80 ^o^C, 6 h | 5 | 99 (100)^a^ | Present study |

^a^Conversion percentage was determined *via* GC-MS.

**Recyclability tests**

**

**

**Figure S7.** SEM image of recovered *h*-BN@APTES@BP@Cu catalyst.

**Figure S8.** XRD spectrum of recovered *h*-BN@APTES@BP@Cu catalyst.

**GC-MS spectra**

**Figure S9.** GC spectrum of 5-Phenyl-1*H*-tetrazole, Table 1, Entry 1.

**
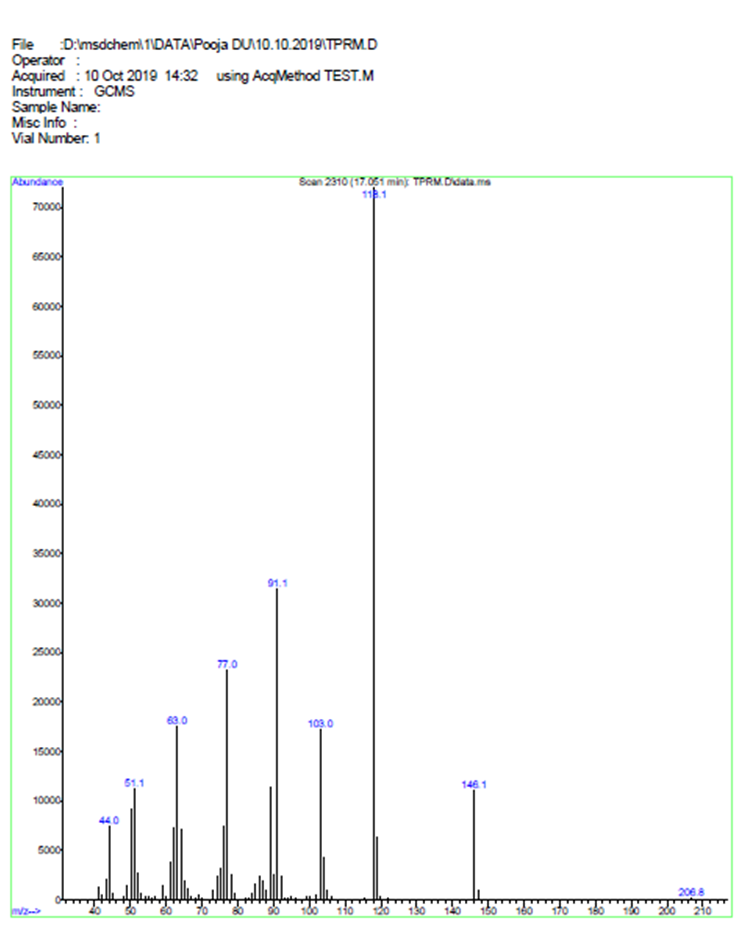
**

**Figure S10.** MS spectrum of 5-Phenyl-1*H*-tetrazole, Table 1, Entry 1.

**Figure S11.** GC spectrum of 5-(4-Cyanophenyl)-1*H*-tetrazole, Table 1, Entry 2.

**
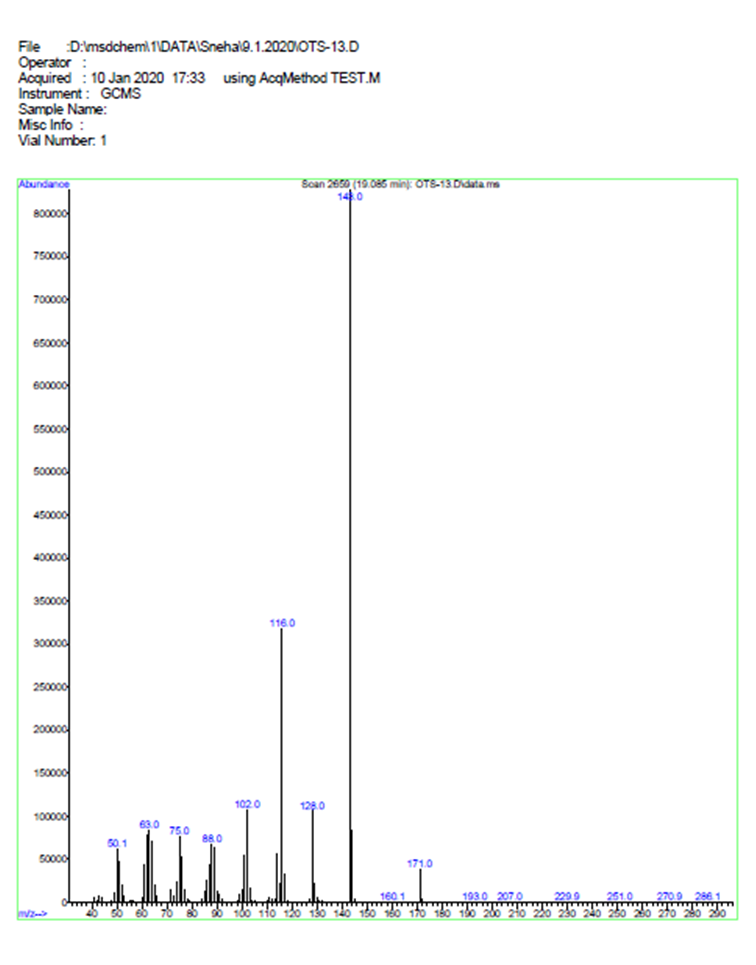
Figure S12.** MS spectrum of 5-(4-Cyanophenyl)-1*H*-tetrazole, Table 1, Entry 2.

**Figure S13.** GC spectrum of 5-(4-Nitrophenyl)-1*H*-tetrazole, Table 1, Entry 3.

**
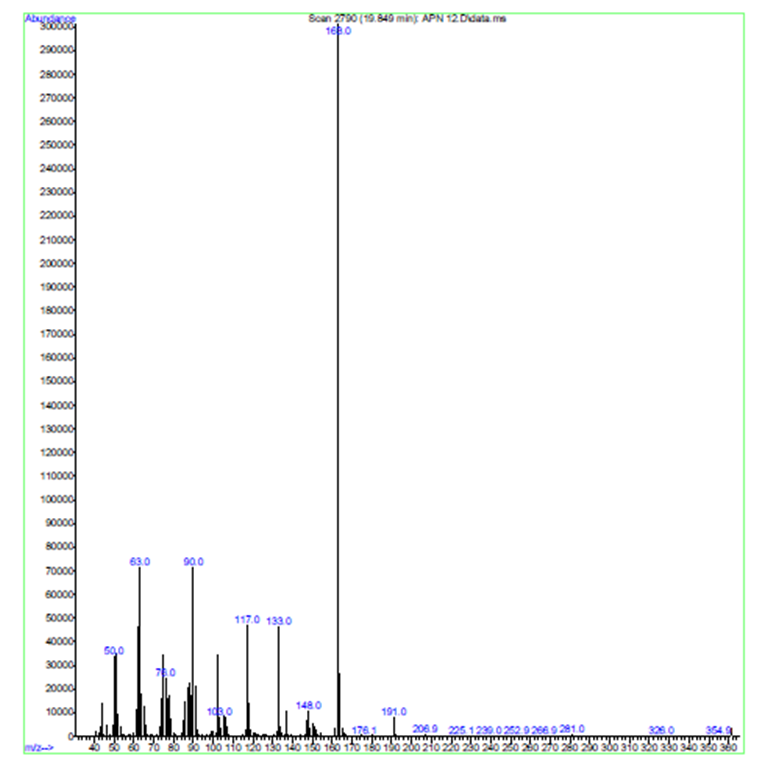
**

**Figure S14.** MS spectrum of 5-(4-Nitrophenyl)-1*H*-tetrazole, Table 1, Entry 3.

**Figure S15.** GC spectrum of 5-(2-Chlorophenyl)-1*H*-tetrazole, Table 1, Entry 4.

**
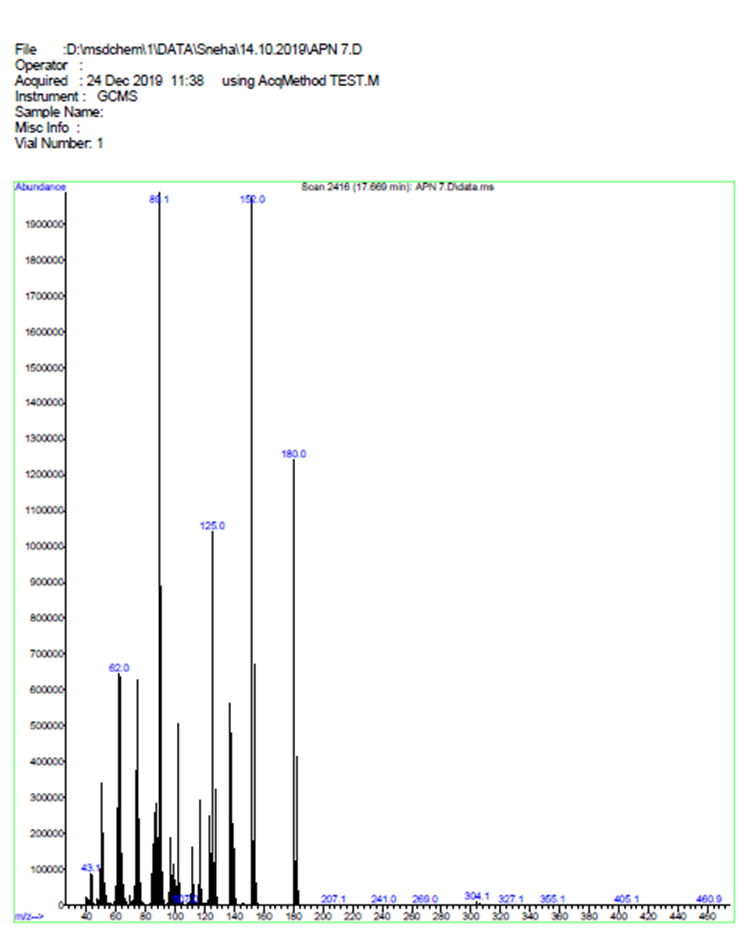
**

**Figure S16.** MS spectrum of 5-(2-Chlorophenyl)-1*H*-tetrazole, Table 1, Entry 4.

**Figure S17.** GC spectrum of 5-(4-Methylphenyl)-1*H*-tetrazole, Table 1, Entry 5.

**
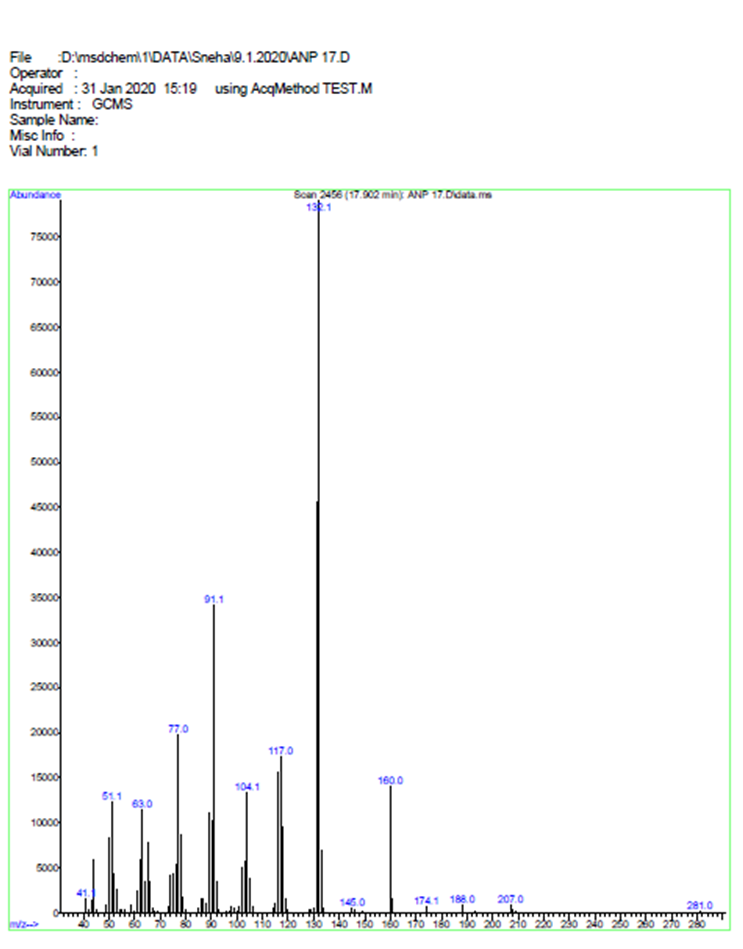
**

**Figure S18.** MS spectrum of 5-(4-Methylphenyl)-1*H*-tetrazole, Table 1, Entry 5.

**Figure S19.** GC spectrum of 5-(4-Methoxyphenyl)-1*H*-tetrazole, Table 1, Entry 6.

**
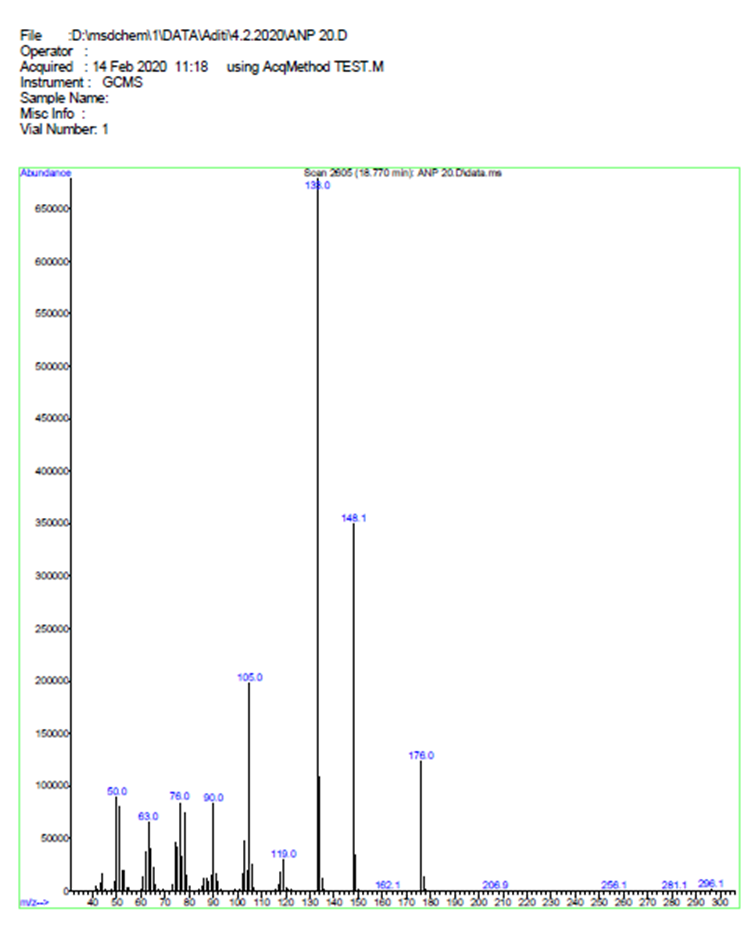
**

**Figure S20.** MS spectrum of 5-(4-Methoxyphenyl)-1*H*-tetrazole, Table 1, Entry 6.

**Figure S21.** GC spectrum of 5-(3-Chlorophenyl)-1*H*-tetrazole, Table 1, Entry 7.

**
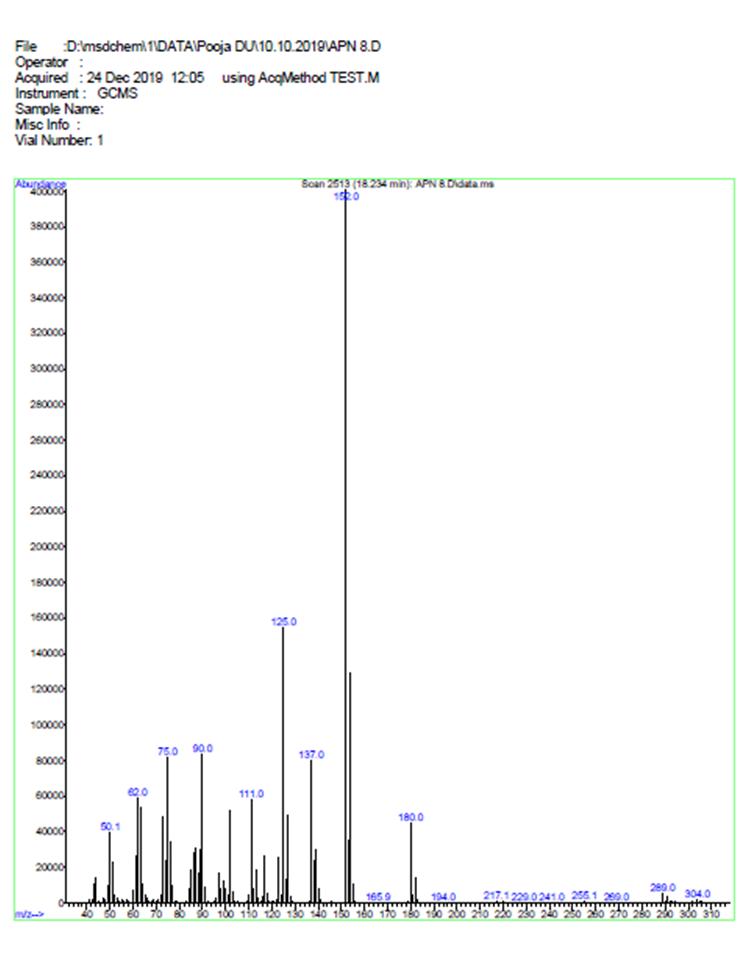
**

**Figure S22.** MS spectrum of 5-(3-Chlorophenyl)-1*H*-tetrazole, Table 1, Entry 7.
